# Depression in mothers at four weeks and twenty-four weeks postpartum: a study on the prevalence and selected risk factors in Sri Lanka

**DOI:** 10.1186/s12884-026-09481-8

**Published:** 2026-06-22

**Authors:** Aththanayaka Liyanage Devika Mihirani Aththanayaka, Piyanjali de Zoysa

**Affiliations:** 1 Faculty of Graduate Studies, University of Colombo, Colombo, Sri Lanka; 2 Faculty of Medicine, University of Colombo, Colombo, Sri Lanka

**Keywords:** Postpartum, Depression, Mothers, Prevalence, Risk factors, Sri Lanka

## Abstract

**Background:**

Although Sri Lanka has shown a significant improvement in maternal health care indicators during the past few decades, PPD could be identified as a significant public health concern. The main purpose of the current study was to assess the most recent status of PPD in Sri Lanka in relation to its prevalence and selected risk factors.

**Methods:**

A population-based quantitative study was conducted among 401 mothers from 18 maternal clinics in the Colombo district, Sri Lanka. The cross-culturally validated Sinhala translation of the Edinburgh Postnatal Depression Scale (EPDS), with a cut-off score of nine or more, was used to estimate the prevalence of PPD. Two questionnaires were designed specifically for the present study to collect data on sociodemographic factors and to extract data from the pregnancy record card.

**Results:**

The prevalence of postpartum depression (PPD) was 29.2% (95% CI 21.6–36.8) at four weeks postpartum and 18.0% (95% CI 13.2–22.8) at twenty-four weeks postpartum. Multivariable analysis identified maternal age 20–24 years (AOR 9.1, 95% CI 1.5–57.2, *p* < 0.05), age 25–34 years (AOR 2.8, 95% CI 1.3–6.4, *p* < 0.05), having three children (AOR 7.9, 95% CI 0.4–44.7, *p* < 0.05), maternal education at Ordinary Level (AOR 4.9, 95% CI 1.2–21.0, *p* < 0.05), maternal history of psychiatric illness (AOR 1.31, 95% CI 1.12–1.55, *p* < 0.01), and abnormalities in the baby (AOR 3.7, 95% CI 1.5–9.1, *p* < 0.01) as factors significantly associated with PPD. In contrast, higher paternal education (AOR 0.3, 95% CI 0.1–0.8, *p* < 0.05) and was associated with lower odds of PPD. Mothers who did not attend prenatal care sessions had markedly higher odds of PPD (AOR 38.1, 95% CI 6.5–222.3, *p* < 0.001).

**Conclusion:**

This study indicates that PPD is highly prevalent among mothers residing in the Colombo district of Sri Lanka. Screening mothers for PPD at various postpartum periods and a multidisciplinary team approach could be introduced to better manage this condition.

## Background

Postpartum depression (PPD) is the most common complication among women following delivery and, as such, represents a considerable public health concern affecting women and their families [1, 2]. PPD is defined as a major depressive episode that typically begins during pregnancy or four weeks after delivery, and symptoms include depressed mood; markedly diminished pleasure in activities; decreased or increased appetite; insomnia or hypersomnia; psychomotor agitation or retardation; fatigue or loss of energy; feelings of worthlessness or guilt; difficulties in concentration; and recurrent thoughts of suicide [3].

PPD has notable effects on the mother–infant relationship, child growth, and development [4]. Furthermore, untreated PPD can have adverse long-term effects on the mother because an episode of PPD can be a precursor of chronic recurrent depression [2]. According to previous studies, suicide is the leading cause of maternal death, and psychiatric diagnoses can be identified in maternal suicides,among them, PPD is the most common [5]. Additionally, PPD can deleteriously affect marital and family relationships.

Approximately one in seven women worldwide experience PPD [6]. In South Asia, the prevalence of PPD ranges from 5 to 49%. Previous studies conducted in Sri Lanka indicate a high prevalence of PPD, which varies from 7.8% to 32.1% [7, 8]. Furthermore, previous research has shown an effect of time since delivery on the prevalence rates of PPD [8]. For example, a study reported that the prevalence of PPD in Sri Lanka was 15.5% at ten days and 7.8% at four weeks postpartum [8]. Moreover, the literature highlights a significant increase in the prevalence rate of PPD during the COVID-19 pandemic [9]. Studies conducted in Sri Lanka have reported increased levels of depressive symptoms among pregnant women during the pandemic. Although these findings primarily relate to antenatal mental health, depression during pregnancy is a well-established risk factor for PPD [10]. In addition, pandemic-related stressors such as economic hardship, social isolation, and disruptions to healthcare services may have further increased the risk of postnatal mental health problems, including PPD [11]. However, there is a paucity of studies specifically examining PPD in Sri Lanka during the COVID-19 period, highlighting a significant gap in the literature.

As a significant step in the field of maternal health in Sri Lanka, in 2012, the validated Sinhala translation of the Edinburgh Postnatal Depression Scale (EPDS) was introduced into routine practice in maternal clinics in Sri Lanka [12]. The Tamil version of the EPDS (validated in Tamil Nadu, India, [13]) was also introduced to the Tamil-speaking population in the country. Since then, all mothers have been routinely screened once, at maternal and child health clinics, for postpartum depression four to six weeks after delivery via the Sinhala or Tamil versions of the EDPS. Because of this more systematic program, there may be some changes in the prevalence rate of PPD at present, as compared to previous reports [8]. Hence, there is a scarcity of most recent studies on the prevalence of PPD in Sri Lanka.

PPD is influenced by complex interactions between biological, environmental, and psychosocial risk factors rather than by a single cause [2]. A previous history of mental disorders, maternal age, low socioeconomic status, physical illnesses during pregnancy, low birth weight, physical illnesses, and poor night sleeping patterns has been identified as associated with PPD in previous studies conducted in Sri Lanka [8, 14, 15].

Although several studies have examined the prevalence and risk factors of PPD in Sri Lanka, most have focused on a single postpartum time point or pre-pandemic populations. There is limited evidence comparing the prevalence of PPD at different postpartum stages within the same cohort, particularly in the context of the COVID-19 pandemic. Furthermore, recent district-level data, especially from the Colombo district, remain scarce. In addition, despite the introduction of routine EPDS screening in maternal and child health clinics, there is a lack of recently published data evaluating current prevalence trends under this new systematic program. Therefore, the current study aims to address these gaps by assessing both the prevalence and selected risk factors of PPD at four weeks and twenty- four weeks. This allows for a more comprehensive understanding of most recent changes in prevalence and associated risk factors under unique public health conditions.

## Methods

### Study design and participants

A population-based cross-sectional quantitative study was conducted among 401 mothers from 18 maternal and child health clinics in the Colombo district, Sri Lanka, in 2021. The sample size was calculated via the formula

$$S ={\chi }^{2}NP (1-P)/\left[{d}^{2}\left(N-1\right)+{\chi }^{2}P\left(1-P\right)\right]$$

(When: χ2 = the table value of chi-square for 1 degree of freedom at the desired confidence level (3.841); N = the population size; P = the population proportion (0.5); d = degree of accuracy expressed as a proportion (0.05) resulting in a sample size of 381. By keeping 5% nonresponses, a total sample of 401 mothers was selected for the study. The inclusion criteria were mothers at four weeks and twenty-four weeks postpartum and mothers registered in the relevant Medical Officer of Health (MOH) area clinics in the Colombo district. The exclusion criteria were mothers whose postpartum period was less than four weeks, those whose postpartum period was between four weeks and twenty-four weeks, those whose postpartum period was more than twenty-four weeks mothers who had experienced infant death and mothers with comorbid mental illnesses. Furthermore, mothers who could not read and understand the Sinhala language including Tamil speaking mothers were excluded as the Sinhala validated version of the EPDS was used.

The Colombo district consists of 18 MOH areas, all of which were included in the study. A list of maternal and child health clinics held under each MOH area was obtained, and one clinic from each MOH area was selected using a simple random sampling method (lottery method). Accordingly, a total of 18 maternal and child health clinics were selected. Since these maternal and child health clinics provide both postpartum care and child immunization services; the same clinics were used to recruit mothers at four weeks postpartum and those attending for the six-month vaccination at twenty-six postpartum. Participants were recruited using a convenience sampling method based on the availability of the eligible mothers during clinic sessions. A fixed sample size was not assigned to each clinic, and the recruitment was continued until the required sample size was achieved.

### Materials

In the present study, three instruments were utilised: the EPDS, which is a 10-item self-report scale (i.e., "I have blamed myself unnecessarily when things went wrong") with four response categories for each item; a specially designed sociodemographic questionnaire (i.e., maternal age, postpartum period, marital status, and mother’s level of education); and a questionnaire for extracting data from the pregnancy record card (i.e., the mother's medical history and the mothers’ psychiatric illness, if any).

### Statistical analysis

The data were analysed via the Statistical Package for the Social Sciences (SPSS 16).

Descriptive statistical analysis was carried out via calculations for the frequencies and percentages of the categorical variables. The outcome variables were (i) EPDS score (continuous variable) and (ii) presence of postpartum depression (binary variable, defined as EPDS score ≥ 9). The independent variables considered were socio-demographic factors (maternal age, postpartum period, marital status, mother's level of education, father's level of education, if the husband has an occupation, if the participant has an occupation, number of living children, and monthly income), Prenatal factors (the mother’s medical history, the mother’s history of psychiatric illness if any, the mother's attendance at antenatal classes, and the father's attendance at antenatal classes), baby's complications (the baby's birth weight, abnormalities detected in the baby and establishment of breastfeeding at discharge from the hospital). These risk factors were selected based on the previous literature and their contextual relevance to the Sri Lankan setting.

Bivariate analysis was carried out to determine the independence of the categorical variables of depressed and nondepressed mothers via the chi-square test and Fisher’s exact test at the 5% level of significance. Bivariate analysis was conducted using chi-square tests and Fisher’s exact tests to assess associations between categorical independent variables and postpartum depression status at the 5% level of significance. Pearson’s correlation analysis was used to assess relationships between continuous variables and EPDS scores.

Variables that were statistically significant at the bivariate level (p < 0.05) were included in multivariable analyses. Multivariable linear regression analysis was performed to identify factors associated with EPDS scores, while multivariable logistic regression analysis was used to identify predictors of postpartum depression (EPDS ≥ 9). Adjusted odds ratios (AORs) with 95% confidence intervals (CIs) were reported for the logistic regression model. All categorical independent variables were entered into the regression models using dummy coding. For each variable, one category was designated as the reference group based on either the highest frequency or clinical relevance. Specifically, maternal age was categorized into three groups (20–24, 25–34, and 35–45 years) based on clinically meaningful reproductive age categories reported in previous maternal mental health studies [16], with the 35–45 age group used as the reference category. Monthly family income was categorized based on socioeconomic distribution patterns reported in the Sri Lankan Household Income and Expenditure Survey [17] and categorization approaches commonly used in maternal health studies [16, 18]. with the highest income group (> Rs. 50,000) used as the reference category. Number of living children was categorized according to the prior literature [8], with higher parity (≥ 4 children) selected as the reference category. All estimates were interpreted relative to these reference categories.

## Results

A total of 387 participants responded to all the questionnaires, among whom 35.4% were from the four-week postpartum category and 64.6% were from the twenty-four-week category. The participants’ characteristics are shown in Table 1.

**Table 1:** Summary of the characteristics of the sample

Background characteristics	Postpartum period % (n)	
	**4 weeks**	**24 weeks**
Overall	35.4(137)	64.6 (250)
Age		
20–24	4.4 (6)	2.8 (7)
25–34	75.9 (104)	46.8 (117)
35–45	19.7 (27)	50.4 (126)
Marital status		
Married	98.5 (135)	97.6 (244)
Unmarried	0.7 (1)	0.4 (1)
Living together	0.0 (0)	0.4 (1)
Widower/divorced/separated	0.7 (1)	1.6 (4)
Mother's education level		
Primary education only	0.7 (1)	0.0 (0)
Passed Grades 6–10	2.9 (4)	2.4 (6)
Passed O/L or equivalent	26.3 (36)	28.8 (72)
Passed A/L or equivalent	55.5 (76)	51.6 (129)
Degree or above	14.6 (20)	17.2 (43)
Father's education level		
Primary education only	1.5 (2)	0.8 (2)
Passed Grades 6–10	0.7 (1)	2.4 (6)
Passed O/L or equivalent	27.0 (37)	33.6 (84)
Passed A/L or equivalent	51.8 (71)	18.4 (121)
Degree or above	19.0 (26)	14.8 (37)
Mother being employed	50.4 (69)	28.8 (72)
Father being employed	100.0 (137)	98.0 (245)
Family Monthly Income		
Less than Rs.15,000	0.7 (1)	0.4 (1)
Between Rs.15,000 and Rs. 30,000	19.0 (26)	5.6 (14)
Between Rs.30,000 and Rs. 50,000	21.2 (29)	38.4 (96)
More than Rs.50,000	59.1 (81)	55.6 (139)
Number of alive children		
1	45.3 (62)	31.6 (79)
2	36.5 (50)	39.2 (98)
3	14.6 (20)	21.2 (53)
4 or above	3.6 (5)	8.0 (20)
The number of prenatal sessions attended by the mother		
0	74.5 (102)	73.6 (184)
1	0.0 (0)	2.8 (7)
2	1.5 (2)	1.6 (4)
3	24.1 (33)	22.0 (55)
The number of prenatal sessions attended by the father		
0	89.1 (122)	81.6 (204)
1	0.0 (0)	1.2 (3)
2	0.0 (0)	0.4 (1)
3	10.9 (15)	16.8 (42)
Mothers with a history of psychiatry illness	2.4 (6)	8.8(12)
Mothers with a history of physical illness	23.4 (32)	10.0 (25)
Abnormalities detected in baby	31.4 (43)	21.2 (53)
Establishment of breastfeeding	86.9 (119)	85.6 (214)
Baby's weight is less than 2500 g	23.4(32)	12.4(31)

### Sociodemographic factors

The majority of those in the four-week postpartum category were in the 25–34 years age group, whereas the majority of those in the 24-week postpartum category were in the 35–45 years age group. Among the mothers at four weeks postpartum, 98.5% were married, and among the mothers at 24 weeks postpartum, 97.6% were married. Most mothers and fathers were qualified at the Advanced Level or equivalent in both the four-week and twenty-four-week postpartum categories. A total of 50.4% and 28.8% of mothers were employed in two categories, four weeks and twenty-four weeks postpartum, respectively. Furthermore, most fathers from the four-week postpartum category had an occupation, whereas 98% of the fathers had an occupation from the twenty-four-week postpartum category. More than 50% of the participants from both categories had a more than Rs. 50,000 monthly income. In the four-week postpartum category, 45.3% of the participants had one child, and in the twenty-four-week postpartum category, the majority of the participants (39.2%) had two children (Table 1).

### Prenatal factors

More than 70% of the mothers and fathers from both the four-week postpartum and twenty-four-week postpartum categories had not attended the prenatal care sessions.

A total of 23.4% of mothers in the four-week postpartum category and 10% of mothers twenty-four weeks postpartum had a history of a physical illness. Additionally, 2.4% of mothers from the four-week postpartum category and 8.8% of mothers from twenty-four weeks postpartum had a history of psychiatric illness (Table 1).

### Baby’s complications

A total of 31.1% of mothers from the four-week postpartum category and 21.2% of mothers from the twenty-four-week postpartum category had babies with abnormalities.

Approximately 85% of mothers were breastfeeding at both four and twenty-four weeks postpartum, and 23.4% and 12.4% of mothers had babies with low birth weights from four weeks and twenty-four weeks postpartum, respectively (Table 1).

### Prevalence of PPD

Among all the participants, 29.2% (95% CI: 21.6%–36.8%) of mothers from the four-week postpartum category and 18% (95% CI: 13.2%–22.8%) of mothers from the twenty-four-week postpartum category had postpartum depression (Table 2). Mothers screened at four weeks postpartum were more likely to have symptoms of depression than those screened at 24 weeks. Descriptive statistics for the EPDS scores are presented in Table 3.

**Table 2:** Prevalence of specific depressive symptoms according to the Edinburgh postnatal depression scale

Number	EPDs	Postpartum period % (n)	
		**4 weeks**	**24 weeks**
Q1	I have been able to laugh and see the funny side of things		
	As much as I always could	53.3 (73)	79.2 (198)
	Not quite so much	46.0 (63)	12.0 (30)
	Definitely not so much	0.7 (1)	8.8 (22)
Q2	I have looked forward with enjoyment to things		
	As much as I ever did	65.0 (89)	75.6 (189)
	Rather less than I used to	34.3 (47)	14.8 (37)
	Definitely less than I used to	0.7 (1)	8.8 (22)
	Hardly at all	0.0 (0)	0.8 (2)
Q3	I had blamed myself unnecessarily when things went wrong		
	yes, most of the time	1.5 (2)	6.4 (16)
	Yes, some of the time	67.9 (93)	51.6 (129)
	Not very often	2.2 (3)	0 (0)
	No, never	28.5 (39)	42.0 (105)
Q4	I have been anxious or worried for no good reason		
	No, not at all	30.7 (42)	67.2 (168)
	Hardly ever	17.5 (24)	4.4 (11)
	Yes, sometimes	51.8 (71)	25.2 (63)
	Yes, very often	0.0 (0)	3.2 (8)
Q5	I have felt scared or panicky for no very good reason		
	Yes, quite a lot	0.0 (0)	4.4 (11)
	Yes, sometimes	30.7 (42)	30.0 (75)
	No, not much	27.7 (38)	2.8 (7)
	No, not at all	41.6 (57)	62.8 (157)
Q6	Things have been getting on top of me		
	yes, most of the time	5.8 (8)	11.2 (28)
	Yes, sometimes	16.8 (23)	10.0 (25)
	No, most of the time	27.0 (37)	11.2 (28)
	No, not at all	50.4 (69)	67.6 (169)
Q7	I have been so unhappy that I have had difficulty sleeping		
	yes, most of the time	5.8 (8)	1.2 (3)
	Yes, sometimes	22.6 (31)	6.8 (17)
	Not very often	21.2 (29)	10.0 (25)
	No, not at all	50.4 (69)	82.0 (205)
Q8	I have felt sad or miserable		
	yes, most of the time	5.8 (8)	0.0 (0)
	Yes, quite often	19.7 (27)	16.8 (42)
	Not very often	22.6 (31)	12.8 (32)
	No, not at all	51.8 (71)	70.4 (176)
Q9	I have been so unhappy that I have been crying		
	yes, most of the time	0.7 (1)	1.2 (3)
	Yes, quite often	12.4 (17)	9.2 (23)
	Only occasionally	22.6 (31)	14.4 (36)
	No, never	64.2 (88)	75.2 (188)
Q10	The thought of harming myself has occurred to me		
	Yes, quite often	0.0 (0)	0.4 (1)
	Sometimes	0.0 (0)	5.6 (14)
	Hardly ever	9.5 (13)	2.4 (6)
	Never	90.5 (124)	91.6 (229)
	EPDS Total Score ≥ 9	29.2 (40)	18 (45)
	Mean EPDS score (95% CI)	7.01	4.76

**Table 3:** Descriptive statistics for the EPDS scores

	**EPDS scores**	
	**4 weeks postpartum**	**24 weeks postpartum**
Mean	7.01	4.76
Median	8.00	3.00
Std. Deviation	4.73	4.84
Range	16	20
Minimum	0	0
Maximum	16	20

### Associated factors of PPD

Table 4 shows the associations between risk factors and EPDS scores.

**Table 4:** Relationships of the potential predictors with EPDS scores and PPD (adjusted analyses)

Background Characteristics	Multivariable Linear Regression	Multivariable Logistic
	EPDS Scores	EPDS Scores ≥ 9
	Beta Coefficient	AOR (95% CI)
Age		
35–45 (ref)		1.00
20–24	0.177***	9.1 (1.5–57.2) *
25–34		2.8 (1.3–6.4)
Mother's education level		
Degree or above (ref)		1.00
Passed Grades 6–10	−0.13	6.0 (0.6–60.0)
Passed O/L or equivalent		4.9 (1.2–21.0) *
Passed A/L or equivalent		2.4 (0.8–7.3)
Mother being employed		
Yes (ref)		1.00
No	−0.081	
Father's education level		
Degree or above (ref)		1.00
Primary education only	0.062	
Passed Grades 6–10		1.3 (0.1–15.3)
Passed O/L or equivalent		0.3 (0.1–1.1)
Passed A/L or equivalent		0.3 (0.1–0.8) *
Father being employed		
Yes (ref)		1.00
No	−0.027	
Family Monthly Income		
≥ Rs.50,000 (ref)		1.00
Less than Rs.15,000	−0.029	
Between Rs.15,000 and Rs. 30,000		
Between Rs.30,000 and Rs. 50,000		
Number of alive children		
≥ 4 (ref)		1.00
1		0.9 (0.2–4.7)
2		3.1 (0.5–17.5)
3	0.187***	7.9 (.4–44.7) *
The number of prenatal sessions Attended by the mother		
≥ 3 (ref)		1.00
0	0.178***	38.1 (6.5–222.3) ***
2		5.6 (0.2–136.9)
The number of prenatal sessions attended by the father		
≥ 3 (ref)		1,00
0	0.165***	0.6 (0.1–4.7)
Abnormalities detected in baby		
No (ref)		1.00
Yes	0.136***	3.7 (1.5–9.1) **
Establishment of breastfeeding		
Yes (ref)		1.00
No	0.156***	0.2 (0.1–0.6) **
Mother has a physical illness history		
No (ref)		1.00
Yes	−0.004	0.96 (0.49–1.88)
Mother has a psychiatric illness history		
No (ref)		
Yes	0.273***	1.31 (1.12–1.55) **
Weight of the baby category		
No (ref)		1.00
Yes	−0.4	1.23 (0.63–2.43)

^***^ < 0.001

^**^ < 0.01

^*^ < 0.05

Table 4 presents the adjusted associations between selected sociodemographic, prenatal, and clinical factors with EPDS scores and postpartum depression (EPDS ≥ 9). Significant risk factors included younger maternal age, higher parity (three children), lack of maternal prenatal care attendance, maternal psychiatric illness, and infant abnormalities, while higher paternal education and successful breastfeeding showed protective effects.

### Sociodemographic factors

The associations of sociodemographic factors with EPDS scores were tested via multivariate linear regression at the 5% significance level. EPDS scores were significantly higher among younger mothers, particularly those aged 20–24 years (β = 0.177, *p* < 0.001) and parity level (β = 0.187, *p* < 0.001). Additionally, EPDS scores were likely to be lower if the partner was employed (β = −0.0270, *p* > 0.001). The presence of postpartum depression was associated with delivery age being between 20 and 24 years (AOR = 9.1, 95% CI 1.5–57.2), having three children (AOR = 7.9, 95% CI 0.4–44.7), having a mother’s education level being O/L qualified (AOR = 4.9, 95% CI 1.2–21.0), and having a father’s educational level being A/L or equivalent (AOR = 0.3, 95% CI 0.1–0.8).

### Prenatal factors

Mothers who did not attend prenatal sessions (β = 0.178, *p* < 0.001) were likely to have higher EPDS scores. EPDS scores were positively related to mothers’ previous history of psychiatric illness (β = 0.273, *p* < 0.001). Mothers who did not attended prenatal care sessions (AOR = 38.1, 95% CI = 6.5–222.3) had significant higher odds of PPD.

### Baby complications

The EPDS scores were positively related to abnormalities detected in the baby (β = 0.136, *p* < 0.001) and the establishment of breastfeeding (β = 0.156, *p* < 0.001). Hence, abnormalities detected in babies (AOR = 3.7, 95% CI 1.5–9.1) and the establishment of breastfeeding (AOR = 0.2, 95% CI 0.1–0.6) were associated with the presence of PPD.

## Discussion

The prevalence of PPD identified in the current study was 29.2% and 18% among mothers at four weeks and twenty-four weeks postpartum, respectively. It is within the prevalence rates indicated in certain other studies [16, 19]. Thus, the results of the current study seem to be higher than those of the most recent study conducted in Sri Lanka, which indicated that the prevalence rates ranged from 7.8% to 15.5% among mothers at ten days and one month postpartum, respectively [8]. This change in the prevalence of PPD could be explained by the findings of studies conducted during the COVID-19 pandemic, as the literature shows a significant increase in the worldwide prevalence rates during the COVID-19 pandemic [9, 20, 21]. For example, a study reported a global prevalence of PPD of 34% during the COVID-19 pandemic [20]. This prevalence rate is much higher than the global pooled prevalence rate of 17%, as indicated by several previous studies conducted during the non-pandemic period [22, 23]. The present study's data were collected during the third wave of the COVID-19 pandemic. The literature shows that pregnant and postpartum mothers are likely to experience sadness, worry, fear, anger, annoyance, frustration, guilt, helplessness, and nervousness when faced with public health emergencies such as pandemics due to the closure of schools and businesses and social distancing, quarantine, and economic fallout [24–27]. Considering the factors mentioned above, the higher prevalence rates indicated by the current study could be justified.

Moreover, the changes in the prevalence of PPD in the current study can be attributed to factors such as the different cut-off points on the EPDS, sampling strategies, study populations, and study settings. For example, a study that focused on mothers at ten days and one month postpartum from two maternal clinics reported PPD prevalence rates of 7.8% and 15.5%, respectively [8], by using a cut-off score for the EPDS of ten or more, which was higher than the validated value. Thus, the use of the validated cut-off score of nine or more for screening for PPD in the current study may have resulted in higher prevalence rates.

However, the prevalence of PPD worldwide has increased during the COVID-19 pandemic [9, 20, 21], and the current study was also conducted during the pandemic, which indicated a higher prevalence rate than did previous studies.

According to the findings of the present study, the factors associated with PPD included (i) sociodemographical factors such as the mothers' age being between 20 and 24 years, which means that younger mothers tend to have higher scores on the EPDS, suggesting a greater risk of experiencingPPD, where younger age at delivery may be a risk factor for PPD; (ii) parity level, which suggests that mothers with three living children tend to have higher EPDS scores, indicating a higher risk of postpartum; (iii) parents' education level, which suggests that mothers who are ordinary level qualified are significantly more likely to experience PPD than those with higher education levels and the potential influence of education on PPD risk; and (iv) prenatal factors such as a previous history of psychiatric illness, which suggests that mothers with a previous history of psychiatric illness are more likely to have higher EPDS scores, indicating an increased risk of postpartum depression, which emphasises the importance of recognising and providing appropriate support to mothers with a history of psychiatric conditions during the postpartum period. (v) Baby complications such as abnormalities detected in the baby are associated with higher EPDS scores in mothers, indicating an increased risk of PPD. This emphasised that babies’ complications contribute to the risk of PPD. Attendance at prenatal clinics was identified as a protective factor, suggesting that mothers who attended prenatal care sessions were significantly more likely to report experiencing PPD than those who did not attend such sessions. This suggests the potential benefits of prenatal care sessions in reducing the risk of PPD. The findings of the current study regarding these risk factors are consistent with those of certain worldwide studies and studies conducted in Sri Lanka [16, 28–33].

The current study indicated that 70% of parents do not attend prenatal care sessions. This could be due to the challenges of health care services during the COVID-19 pandemic [9]. The literature shows that mothers who live in urban areas have lower participation in health education programs during pregnancy [34]. Mothers' illnesses before and after pregnancy, conflicts with their husbands, use of harsh words by their husbands, physical abuse during pregnancy, death of a friend, normal vaginal delivery, and baby's complications, such as low birth weight, illness, poor sleeping patterns, and prematurity, are the other associated factors of PPD indicated by other studies conducted in Sri Lanka [15]. When the results regarding risk factors are compared with those of other studies, factors related to babies' complications and mothers' previous mental illness can be identified as well-established risk factors that need attention in the Sri Lankan maternal mental health care field.

Notably, in the current study, the prevalence of PPD appeared to be greater at four weeks postpartum than at twenty-four weeks postpartum.

The prevalence of PPD appears to be more common early than late after birth. This finding is supported by the results of previous studies [8, 35–37]. The higher prevalence rate at four weeks postpartum may be associated with abrupt hormonal changes immediately following childbirth, such as changes in estragon and progesterone levels [38, 39] and new psychosocial changes in a mother's life because of adapting to the new role of parenthood [40].

## Limitations

The limitations of the study are related mainly to the methodology. Although a simple random sampling method was used to select the maternal and child health clinics, the participants were selected via the convenient sampling method, which may have resulted in selection bias. The prevalence of PPD could have been overestimated when the EPDS was used, as self-reported instruments usually report a higher prevalence rate [2, 41, 42] than do diagnoses established through clinical interviews. The study design of the current study can analyse only associations and not causation. In addition, categorizing maternal age may have reduced the precision of the analysis compared to treating it as a continuous variable. Also, the wide confidence intervals observed for some variables, such as prenatal care attendance, may be due to small subgroup sizes and should be interpreted with caution. Although several relevant variables were included, not all potential risk factors for postpartum depression were assessed in this study. Furthermore, although PPD screening is routinely conducted in maternal clinics in Sri Lanka at four to six weeks postpartum, systematically published data from these programs are limited. Therefore, direct comparisons with routine screening data were also not feasible. Additionally, in line with the objectives of the current study, an analysis of PPD levels was not conducted, as the EPDS primarily serves as a screening tool, and its design and purpose do not allow it to be used for a detailed analysis of PPD severity. Finally, as the Colombo district was purposely selected, the results might not be fully generalizable to other parts of Sri Lanka.

## Conclusion

Although Sri Lanka has shown a significant improvement in maternal health care indicators during the past few decades, PPD could be identified as a significant public health concern. According to the results, the prevalence of PPD was 29.2% and 18% among mothers at four weeks and twenty-four weeks postpartum, respectively. Additionally, sociodemographic factors, such as mothers' age between 20 and 24 years, parity level, parents' education level, prenatal factors, such as history of psychiatric illness, and the number of prenatal clinic visits and baby's complications, such as abnormalities detected in the baby were identified as risk factors associated with PPD. These findings show that PPD is highly prevalent among mothers residing in the Colombo district compared to previously reported prevalence rates in Sri Lanka and similar settings. In addition, the prevalence is greater among mothers at four weeks than among those at twenty-four weeks postpartum. Moreover, a multidisciplinary team approach involving obstetric, psychiatric, midwife, psychologist is essential for effective management of PPD. In this collaborative approach each professional plays a distinct yet integrated role to provide holistic care. Integrating PPD screening by using EPDS at different time points in postpartum rather than screening only in four weeks postpartum and during pregnancy could also be recommended as an essential step in the early detection, treatment, and prevention of PPD in Sri Lanka. As well as, EPDS can be administered in various settings, including maternal clinics, home visits, paediatric clinics, and obstetric clinics. Conducting awareness programs on psychological wellbeing of mothers is a vital component of preventing PPD since these programs would promote self-care practices, discuss stress management techniques, and equip mothers with the tools that they need to cope with the demands of new motherhood. Since prenatal care sessions provide support and information for mothers and their partners about mothers' physical and mental health during pregnancy and postpartum and essential baby care strategies to adapt to parenthood, a systematic program to increase the attendance of mothers' and fathers' prenatal care sessions should be implemented. Furthermore, conducting these sessions online is recommended specially during pandemics or disaster situations. Moreover, further studies should aim to develop a culturally appropriate risk assessing instrument including biopsychosocial risk factors for identification of high-risk mothers in the community settings. PPD needs the urgent attention of mental health care professionals for early detection, treatment, and prevention to decrease its effects on mothers, babies, their families, and society.

## Data Availability

The data that support the findings of this study are available from the corresponding author upon reasonable request.

## Abbreviations

AOR: Adjusted Odds Ratio
CI: Confidence Interval
COVID-19: Coronavirus Disease
EPDS: Edinburgh Postnatal Depression Scale
MOH: Medical Officer of Health
PPD: Postpartum Depression
